# Comparative Outcomes of Bilateral Versus Unilateral Total Knee Arthroplasty: A Big Data Analysis

**DOI:** 10.3390/healthcare13091033

**Published:** 2025-04-30

**Authors:** David Maman, Daniel Dumov, Maneesh Nandakumar, Batia Litmanowicz, Daniel Shpigelman, Linor Fournier, Yaniv Steinfeld, Yaniv Yonai, Yaron Berkovich

**Affiliations:** 1 Carmel Medical Center, Haifa 3436212, Israel; linorfournier@gmail.com (L.F.); yanivsteinfeld@gmail.com (Y.S.); yanivyonai@gmail.com (Y.Y.); yaron.berkovich@gmail.com (Y.B.); 2 Faculty of Medicine, Technion Israel Institute of Technology, Haifa 2611001, Israel; danieldumov1@gmail.com (D.D.); batialitma@gmail.com (B.L.); 3 School of Medicine and Public Health, University of Newcastle, Callaghan, NSW 2308, Australia; maneesh.nan@gmail.com; 4 Logan Hospital, Brisbane, QLD 4131, Australia; 5 Faculty of Medicine, Lithuanian University of Health Sciences, 44307 Kaunas, Lithuania; daniel4ik@gmail.com

**Keywords:** bilateral total knee arthroplasty, unilateral total knee arthroplasty, perioperative complications, hospital costs, length of stay, big data, elective surgery, risk stratification

## Abstract

**Background:** Bilateral total knee arthroplasty (B-TKA) is a surgical option for patients with bilateral osteoarthritis, offering potential efficiency and cost advantages but with increased perioperative risk. **Methods:** We conducted a retrospective analysis of 2,299,979 elective TKA cases from the Nationwide Inpatient Sample (2016–2019). Propensity score matching (PSM) was used to compare 83,980 B-TKA patients with matched unilateral TKA (U-TKA) patients. Outcomes included in-hospital mortality, complications, length of stay, and hospital charges. **Results:** B-TKA patients had higher rates of complications such as deep vein thrombosis (OR 1.798) and pulmonary embolism (OR 1.883), longer hospital stays (3.03 vs. 2.49 days), and higher charges (USD 83,639 vs. USD 59,215; all *p* < 0.001). **Conclusions:** Although B-TKA is associated with increased perioperative risk, it may offer logistical and economic advantages in well-selected patients. These findings support the need for risk stratification in surgical decision-making.

## 1. Introduction

Total knee arthroplasty (TKA) is an effective surgical procedure for alleviating pain and restoring function in patients with severe knee osteoarthritis. Unilateral TKA (U-TKA) involves replacing a single knee joint and is associated with shorter surgery times, lower costs, and fewer complications than bilateral TKA (B-TKA) [1,2,3]. B-TKA, which replaces both knee joints, can be performed simultaneously or in stages. While simultaneous B-TKA has higher initial costs and complication risks, it may reduce the overall hospitalization period and cumulative costs compared to two separate U-TKAs [2,3,4]. From 2007 to 2016, approximately 276,194 B-TKA and 5,528,429 U-TKA procedures were performed in the United States [5]. Advances in surgical techniques and perioperative care have made B-TKA a viable option for patients with bilateral knee disease. Despite several prior studies comparing bilateral and unilateral TKA, including those using NSQIP and single-center data, few have focused on national, inpatient charge-based outcomes with broad comorbidity balancing. This study addresses this gap by analyzing economic and clinical outcomes using the largest U.S. inpatient database available, NIS, and leveraging robust propensity score matching over a four-year span.

The decision between U-TKA and B-TKA depends on patient-specific factors such as overall health, comorbidities, and the severity of bilateral knee osteoarthritis. Simultaneous B-TKA is typically considered for younger, healthier patients with severe bilateral disease and minimal comorbidities. Candidates may include those under 75 years of age and patients with significant varus malalignment of the contralateral knee [6].

Immediate postoperative outcomes differ significantly between B-TKA and U-TKA in terms of cost, hospital stay, and complications. B-TKA is associated with higher hospital costs [7] and a greater likelihood of discharge to rehabilitation facilities (70% vs. 32%) [7]. Additionally, B-TKA has increased rates of pulmonary embolism (0.5% vs. 0.2%), stroke (0.3% vs. 0.1%), acute blood loss anemia (18.5% vs. 9.0%), blood transfusion (38.0% vs. 5.0%), and 90-day readmission (7.5% vs. 5.5%) compared to U-TKA [8]. The overall complication rate for B-TKA is approximately 12.2%, compared to 8.2% for U-TKA [9].

Despite growing research on B-TKA, comparative analyses with U-TKA remain limited due to small sample sizes and single-center studies. Big data analytics provides a solution by leveraging large, multi-institutional datasets to improve risk stratification, predict complications using machine learning, and directly compare surgical approaches. For instance, Warren et al. used the National Surgical Quality Improvement Program (NSQIP) to show that even the healthiest B-TKA patients had a more than threefold increase in complications compared to U-TKA [10]. Similarly, Odum and Springer analyzed Nationwide Inpatient Sample (NIS) data, finding higher odds of minor (OR 1.51) and major (OR 1.30) complications in B-TKA [11]. By utilizing large-scale datasets, big data enhances the understanding of complication risks, optimizes patient selection, and improves surgical decision-making.

Compared to single-center studies, national administrative databases such as the NIS provide broader generalizability and the statistical power to detect rare complications. While previous big data studies have evaluated complication rates, our analysis expands on this work by including a broader timespan, focusing on inpatient charges, and highlighting a surprising trend—lower infection-related complications in B-TKA despite longer hospital stays and higher transfusion rates. This was unexpected, as longer hospitalization typically correlates with higher infection risk.

From a health economics perspective, simultaneous B-TKA may offer improved cost–utility by consolidating hospital admissions and anesthesia exposures, potentially reducing indirect costs. Cost–benefit tradeoffs must be interpreted within each health system’s resource capacity and patient risk profile. Our use of the NIS dataset builds on prior propensity score-matched studies by including a broader national sample and capturing both hospital-level variables and payer type, which are underexplored in the NSQIP-based literature.

This study aims to compare immediate postoperative outcomes, including costs, hospital stay, and complication rates, between B-TKA and U-TKA using a nationwide inpatient database. By leveraging big data, we seek to provide definitive insights into the benefits and risks of each approach, ultimately informing clinical decision-making. We hypothesize that while B-TKA presents greater perioperative risks, it may offer comparable or better overall outcomes in hospital costs and length of stay. This research will contribute to the growing evidence guiding TKA surgical selection, improving patient care, and optimizing resource utilization.

### Research Question

In patients undergoing TKA, how do immediate postoperative outcomes—including hospital costs, length of stay, and complication rates—differ between B-TKA and U-TKA based on nationwide inpatient data?

## 2. Methods

### 2.1. Dataset Acquisition and Inclusion Criteria

We conducted a retrospective analysis using the Nationwide Inpatient Sample (NIS), a comprehensive, publicly accessible database encompassing inpatient hospitalizations across the United States. From this dataset, 2,299,979 patients undergoing TKA were identified, with 83,980 classified as bilateral procedures (B-TKA), representing 3.65% of all cases. The analysis was limited to elective TKA admissions, selected based on relevant ICD-10 procedure codes specific to knee arthroplasty. Healthcare formatting guidelines were followed.

### 2.2. Study Period and Data Source

The data span from 1 January 2016 to 31 December 2019, capturing the most recent information available within the NIS system at the time of the study.

### 2.3. Patient Identification and Exclusions

Patients were identified based on ICD-10 coding related to total knee replacement. The study excluded non-elective admissions and cases where surgery was performed prior to hospital admission. This ensured a focus on elective, primary TKA procedures.

### 2.4. Statistical Analyses

All statistical evaluations were carried out using SPSS version 26, with the significance threshold set at *p* < 0.05. To reduce the impact of selection bias and account for baseline differences in comorbidities, a propensity score matching (PSM) approach was implemented. Propensity score matching was performed using a 1:1 nearest-neighbor algorithm with a caliper width of 0.01. Variables included in the model were age, sex, BMI, primary payer, hospital region, comorbidities (e.g., hypertension, diabetes), and hospital teaching status. This technique allowed for the creation of two well-balanced cohorts—U-TKA and B-TKA with equivalent distributions across key variables. After matching, each group consisted of 83,980 patients with similar demographic profiles, insurance types, and hospital-related characteristics, ensuring more accurate and meaningful comparisons between the two procedures. A caliper width of 0.01 was chosen to ensure a tight match between groups, minimizing residual bias. After matching, the balance was verified using standardized mean differences (SMD < 0.1 for all variables). Multicollinearity was assessed using variance inflation factors (VIFs), and no significant collinearity was observed among matching variables.

### 2.5. Outcome Measures and Procedure Identification

The primary outcomes included in-hospital mortality, length of stay, total hospital charges, and postoperative complications. Postoperative complications were identified using standardized ICD-10-CM codes (full list available in Supplementary Table S1). Each complication was defined based on primary or secondary diagnosis fields recorded during the inpatient admission: code, ileus, sepsis, blood transfusion, pulmonary edema, blood loss anemia, pulmonary embolism (PE), deep vein thrombosis (DVT), heart failure, acute renal failure, urinary tract infection (UTI), and pneumonia.

### 2.6. Ethical Considerations

This study utilized the NIS, a publicly available, de-identified database provided by the Healthcare Cost and Utilization Project (HCUP) and sponsored by the Agency for Healthcare Research and Quality (AHRQ). In accordance with HCUP data use agreements and U.S. federal regulations, analyses based on de-identified datasets such as the NIS do not constitute human subjects research, as defined by 45 CFR 46.102. Therefore, this study did not require Institutional Review Board (IRB) approval or informed consent. This study was conducted in accordance with the principles of the Declaration of Helsinki and relevant institutional guidelines for the use of de-identified patient data.

## 3. Results

**Figure 1:**
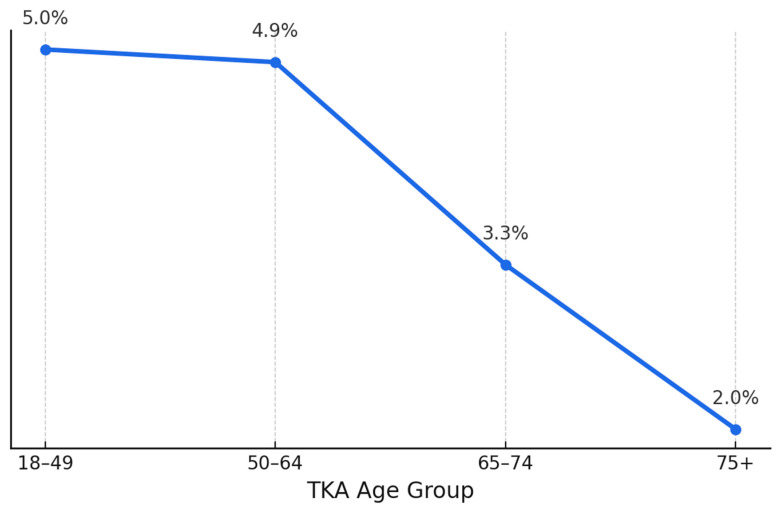
Age-dependent distribution of bilateral total knee arthroplasty proportions compared to unilateral TKA.

As shown in Figure 1, among the 2.3 million TKA procedures identified, 83,980 (3.65%) were bilateral. As shown in Figure 1, younger patients were more likely to undergo B-TKA, with a significant decrease in utilization among those aged 75 and older (*p* < 0.001).

### 3.1. Demographic and Hospital Characteristics of Patients Undergoing Bilateral vs. Unilateral Total Knee Arthroplasty

Table 1 summarizes the demographic and hospital-related differences between groups. B-TKA patients were slightly older, more likely to have private insurance, and more often treated in urban teaching hospitals. Although subgroup analyses by age and insurance type were not performed in this study, the descriptive trends in Table 1 suggest structural disparities in access to B-TKA. These merit further investigation in future studies.

### 3.2. Body Mass Index (BMI) Comparison Between Unilateral and Bilateral Total Knee Arthroplasty Patients

As shown in Table 2, BMI distributions were similar between groups.

### 3.3. Comparison of Comorbidities Between Unilateral and Bilateral Total Knee Arthroplasty Patients

As shown in Table 3, patients selected for B-TKA had significantly fewer comorbidities than those undergoing U-TKA, particularly in terms of hypertension, diabetes, and kidney disease.

### 3.4. Propensity Score-Matched Analysis to Balance Baseline Characteristics

To reduce the risk of selection bias and account for baseline comorbidity variations between patients undergoing U-TKA and B-TKA, a propensity score-matched analysis was conducted. This method ensures that the two groups are statistically equivalent, enhancing the reliability of the comparison and minimizing confounding variables. The results of this propensity score-matched analysis, which include a detailed comparison of demographics, payer information, and comorbidities, are presented in Table 4. No significant differences were observed between the groups across most parameters, underscoring the effectiveness of the matching process and confirming the homogeneity of the patient cohorts.

### 3.5. Comparison of Hospitalization Outcomes in Propensity Score-Matched Cohorts

As shown in Table 5, B-TKA was associated with longer hospital stays, higher total charges, and a slightly increased in-hospital mortality rate.

### 3.6. Odds Ratios of Postoperative Complications in Bilateral TKA Versus Unilateral TKA in a Propensity Score-Matched Cohort

**Figure 2:**
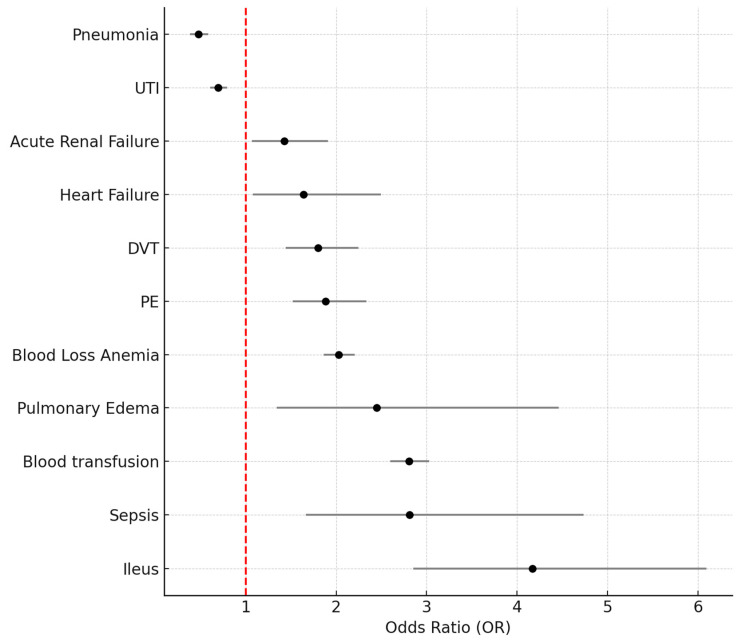
Forest plot summarizing adjusted odds ratios of postoperative complications in bilateral TKA compared to unilateral TKA in a propensity score-matched cohort.

Figure 2 illustrates the adjusted odds ratios (ORs) and 95% confidence intervals (CIs) for key postoperative complications in patients undergoing B-TKA compared to U-TKA in a propensity score-matched cohort. Only complications with statistically significant differences (*p* < 0.05) are presented, emphasizing areas of increased or decreased perioperative risk associated with B-TKA.

Among the complications with lower odds in the B-TKA group, pneumonia demonstrated a significantly reduced risk (OR 0.476, 95% CI: 0.384–0.588, *p* < 0.001), as did urinary tract infections (OR 0.696, 95% CI: 0.609–0.796, *p* < 0.001). These findings may reflect improved preoperative optimization and shorter catheterization duration in carefully selected bilateral cases.

In contrast, B-TKA was associated with elevated risks for several complications. These include the following:

- Acute renal failure (OR 1.429, 95% CI: 1.070–1.907, *p* = 0.015);
- Heart failure (OR 1.640, 95% CI: 1.078–2.494, *p* = 0.021);
- Deep vein thrombosis (DVT) (OR 1.798, 95% CI: 1.441–2.244, *p* < 0.001);
- Pulmonary embolism (PE) (OR 1.883, 95% CI: 1.519–2.335, *p* < 0.001);
- Blood loss anemia (OR 2.026, 95% CI: 1.862–2.204, *p* < 0.001);
- Pulmonary edema (OR 2.451, 95% CI: 1.346–4.462, *p* = 0.003);
- Blood transfusion (OR 2.806, 95% CI: 2.600–3.029, *p* < 0.001);
- Sepsis (OR 2.809, 95% CI: 1.666–4.737, *p* < 0.001);
- Ileus, which had the highest relative risk (OR 4.171, 95% CI: 2.855–6.094, *p* < 0.001).

These findings highlight that although B-TKA is often reserved for healthier individuals, the physiological burden of simultaneous bilateral surgery may still predispose patients to increased systemic complications. These elevated odds should be considered in risk–benefit discussions with surgical candidates.

## 4. Discussion

### 4.1. Main Findings

Our study found that B-TKA represents approximately 4% of all TKA procedures and is associated with higher perioperative risk, including increased odds of thromboembolic complications, blood transfusion, and mortality. However, this elevated risk must be interpreted within the context of potential economic and logistical benefits for selected patient groups.

Our findings indicate that patients who underwent B-TKA represented approximately 4% of all cases. This prevalence is slightly higher than previously suggested in the literature. Earlier studies reported that bilateral procedures are more common than traditionally assumed, with prevalence estimates approaching 5%, reinforcing that B-TKA should be considered a viable option in appropriately selected patients [4,5].

### 4.2. Patient Selection and Health Equity Considerations

The demographic and clinical data (Table 1) show that patients undergoing B-TKA are, on average, slightly older than those undergoing U-TKA, with a mean age of 67.1 years versus 66.1 years (*p* < 0.001). Additionally, the B-TKA group had a lower proportion of females (54% vs. 62%, *p* < 0.001). BMI distribution was similar across both groups (Table 2).

More importantly, B-TKA patients exhibited significantly lower rates of several comorbidities compared to U-TKA patients. Rates of hypertension, dyslipidemia, and diabetes were all lower among B-TKA patients, consistent with the tendency to select healthier individuals for simultaneous surgery. These findings align with previous studies suggesting that optimal candidates for B-TKA typically have controlled diabetes and no significant pulmonary disease, renal insufficiency, or major cardiac conditions, and generally exhibit better preoperative functional status [8,12,13,14,15,16,17,18]. These findings highlight the importance of rigorous patient selection criteria to ensure that only individuals who can physiologically tolerate the increased demands of a bilateral procedure are chosen. Our analysis also identified socioeconomic disparities in access to B-TKA, as patients undergoing the procedure were significantly more likely to be privately insured, while those undergoing U-TKA were more often covered by Medicare. While this variable was controlled in the matched analysis, these trends suggest that institutional or payer-level factors may influence surgical eligibility, with implications for healthcare equity and policy.

### 4.3. Perioperative Risk and Complication Profiles

Despite the favorable baseline characteristics of B-TKA patients, our propensity score-matched analysis revealed increased odds of certain complications (Figure 2) and a higher mortality rate (Table 5). Even after balancing comorbidities, B-TKA was associated with significantly increased risks for thromboembolic events and transfusion. These findings are consistent with prior studies and reflect the physiological burden of simultaneous bilateral surgery. Previous research similarly found that B-TKA is associated with a higher incidence of pulmonary embolism, cardiovascular complications, acute blood loss anemia, and increased mortality, particularly in older adults and those with multiple comorbidities [8,19,20].

These increased risks may be attributed to the greater physiological burden associated with simultaneous knee replacement, including larger surgical wounds, more extensive bone resection, increased cardiac and pulmonary strain, and longer surgical and anesthesia durations. However, it is worth noting that undergoing two separate unilateral procedures may expose patients to cumulative surgical and anesthetic risks, which in some cases could outweigh the perioperative risk of B-TKA.

Additionally, B-TKA requires only one exposure to general anesthesia compared to two separate exposures in staged procedures. This could be particularly beneficial for older adults or those with existing comorbidities, as it reduces the risks associated with repeated intubation, hemodynamic instability, and adverse drug reactions.

### 4.4. Economic Implications and Hospital Resource Utilization

Our analysis indicates that patients undergoing B-TKA experienced significantly longer hospital stays and higher associated charges compared to those undergoing U-TKA (Table 5). This finding aligns with previous studies, which have shown that B-TKA typically requires extended hospitalization due to the need for increased monitoring, enhanced pain control, and delayed mobilization [4,21,22].

However, simultaneous B-TKA may ultimately prove more cost-effective than staged procedures by consolidating preoperative testing, anesthesia, hospitalization, and rehabilitation into a single episode of care. Several studies have reported that B-TKA is associated with reduced overall system costs when compared to two sequential U-TKAs [13,23]. Notably, Franceschetti et al. demonstrated that B-TKA had a shorter cumulative LOS compared to staged procedures, further supporting its potential economic utility [14,15,22]. From a healthcare systems perspective, particularly in resource-limited environments, the reduction in duplicated care episodes may offer substantial logistical and economic advantages if patient selection is rigorously applied.

### 4.5. Decision-Making and Clinical Relevance

Our findings support the importance of personalized surgical planning based on the comorbidity profile, socioeconomic context, and access to postoperative care. Individualized decision-making is essential, especially when balancing the increased risk of B-TKA against its potential for streamlined recovery and cost savings.

While prior studies have proposed risk stratification tools—such as the BTK Safety Score—to assist in identifying suitable candidates for simultaneous procedures [4], these frameworks were not directly applied in our study. Nonetheless, our results strongly support their continued development and use in clinical practice. Future integration of fast-track rehabilitation protocols may help mitigate the prolonged hospital stays associated with B-TKA, improving both efficiency and patient satisfaction.

### 4.6. Limitations

This study is subject to several limitations inherent to the structure of the NIS, which is an administrative database based on ICD-10 coding. Although large and nationally representative, the NIS lacks detailed clinical data such as implant type, surgical technique, intraoperative blood loss, operative duration, anesthesia method, and discharge disposition—all of which can influence outcomes but are unavailable for analysis. The NIS does not permit long-term follow-up of patients. As such, we were unable to evaluate outcomes beyond the index hospitalization, including 90-day readmission, functional recovery, revision surgery rates, or patient-reported outcome measures (PROMs). This limits our ability to assess the sustained clinical value or functional impact of bilateral versus unilateral TKA. Our analysis also does not account for surgeon-specific or hospital-level variation in outcomes. Factors such as surgical volume, team experience, and institutional protocols may affect complication rates and length of stay but were not available in this dataset.

We employed PSM to reduce confounding from baseline differences; however, this method only balances observed variables. Residual confounding due to unmeasured factors such as frailty, physical function, patient motivation, social support, and rehabilitation access may still influence the outcomes.

Despite these limitations, the use of a large, validated national dataset combined with a robust matching methodology enhances both the generalizability and internal validity of our findings.

## 5. Conclusions

B-TKA presents higher perioperative risks compared to U-TKA, including increased rates of thromboembolic events, blood transfusion, and in-hospital mortality. However, it may offer logistical and economic advantages by consolidating recovery into a single hospitalization. Our findings emphasize the importance of careful patient selection, particularly among healthier individuals with fewer comorbidities. Additionally, differences in insurance coverage between B-TKA and U-TKA patients suggest potential disparities in access and cost implications. Future studies should integrate risk stratification tools and explore long-term outcomes to further optimize surgical planning and improve patient care.

## Figures and Tables

**Table 1:** A comparative analysis of U-TKA and B-TKA across age, payer distribution, gender, and hospital type.

Parameter	Unilateral TKA	Bilateral TKA	Significance
Total surgeries (%)	2,215,999	83,980	
Average age (y)	66.1	67.1	*p* < 0.001
Female (%)	62.0%	54.0%	*p* < 0.001
Primary expected payer—Medicare (%)	57.7%	43.0%	*p* < 0.001
Payer—Medicaid (%)	4.3%	3.7%	
Payer—private (%)	34.4%	50.3%	
Payer—self-pay (%)	0.5%	0.7%	
Primary—no charge (%)	0.0%	0.0%	
Primary—other (%)	3.1%	2.3%	
Location/teaching status—rural (%)	10.4%	9.7%	*p* < 0.001
Location/teaching status—urban nonteaching (%)	27.4%	22.8%	
Location/teaching status—urban teaching (%)	62.2%	67.4%	

**Table 2:** Comparative analysis of U-TKA and B-TKA mean BMI.

	Unilateral TKA	Bilateral TKA	Significance
Mean BMI	36.7	35.9	*p* = 0.41
Std. Deviation	4.5	4.5	

**Table 3:** Prevalence of comorbidities in patients who underwent either unilateral TKA or bilateral TKA.

Parameter	Unilateral TKA	Bilateral TKA	Significance
Hypertension (%)	59.7%	55.5%	*p* < 0.001
Dyslipidemia (%)	46.9%	40.8%	*p* < 0.001
Obstructive Sleep Apnea (%)	13.2%	12.8%	*p* = 0.01
Chronic Anemia (%)	5.7%	6.5%	*p* < 0.001
Alcohol Abuse (%)	0.9%	1.1%	*p* < 0.001
Osteoporosis (%)	4.0%	2.7%	*p* < 0.001
Parkinson Disease (%)	0.6%	0.3%	*p* < 0.001
Alzheimer Disease (%)	0.2%	0.1%	*p* < 0.001
Chronic Kidney Disease (%)	7.0%	4.8%	*p* < 0.001
Congestive Heart Failure (%)	1.2%	0.7%	*p* < 0.001
Chronic Lung Disease (%)	6.1%	3.8%	*p* < 0.001
Diabetes Mellitus (%)	22.0%	15.9%	*p* < 0.001
IBD (%)	0.5%	0.5%	*p* = 0.357
Liver Disease (%)	1.3%	1.3%	*p* = 0.819
Obesity (%)	31.1%	30.7%	*p* = 0.040
Fibromyalgia (%)	2.8%	2.2%	*p* < 0.001
Disorders of Thyroid (%)	18.0%	15.4%	*p* < 0.001
History of Myocardial Infarction (%)	3.2%	2.0%	*p* < 0.001
Peripheral Vascular Disease (%)	1.5%	1.0%	*p* < 0.001
History of Cerebrovascular Accident (%)	4.1%	2.3%	*p* < 0.001
Dementia (%)	0.5%	0.3%	*p* < 0.001
Neoplasms (%)	0.9%	1.0%	*p* = 0.479
Neoplasms of Lymphoid and Hematopoietic Tissue (%)	0.4%	0.4%	*p* = 0.634

**Table 4:** Comparison of demographic and clinical data in propensity score-matched cohorts of unilateral TKA and bilateral TKA.

Parameter	Unilateral TKA	Bilateral TKA	Significance
Total surgeries (number)	83,980	83,980	
Average age (years)	63.9	63.9	*p* = 0.90
Female (%)	54.2	54.0	*p* = 0.59
Payer—Medicare (%)	43.0	43.0	*p* = 0.10
Payer—Medicaid (%)	3.7	3.7	
Payer—private (%)	50.3	50.3	
Payer—other (including self-pay) (%)	2.3	2.3	
Location/teaching status—rural (%)	10.0	9.7	*p* = 0.61
Location/teaching status—urban nonteaching (%)	22.7	22.9	
Location/teaching status—urban teaching (%)	67.3	67.4	
Hypertension diagnosis (%)	55.5	55.5	*p* = 0.94
Dyslipidemia diagnosis (%)	40.8	40.8	*p* = 0.85
Sleep apnea diagnosis (%)	12.7	12.8	*p* = 0.59
Chronic anemia (%)	6.3	6.5	*p* = 0.08
Alcohol abuse (%)	1.1	1.1	*p* = 0.82
Osteoporosis (%)	2.6	2.7	*p* = 0.60
Parkinson disease (%)	0.2	0.3	*p* = 0.09
Type 2 diabetes (%)	15.8	15.9	*p* = 0.87
Renal disease (%)	4.7	4.8	*p* = 0.10
Chronic heart failure (%)	0.7	0.7	*p* = 0.46
Chronic lung disease (%)	3.7	3.8	*p* = 0.52
Obesity (%)	30.7	30.7	*p* = 0.11
Fibromyalgia (%)	2.3	2.2	*p* = 0.08
Thyroid disorders (%)	15.3	15.4	*p* = 0.09
History of MI (%)	2.1	2.0	*p* = 0.07
Peripheral vascular disease (%)	1.2	1.0	*p* = 0.07
History of CVA (%)	2.3	2.3	*p* = 0.08
Dementia (%)	0.3	0.3	*p* = 0.09
Peptic ulcer disease (%)	0.3	0.4	*p* = 0.08
Neoplasms (%)	1.0	1.0	*p* = 0.07
Neoplasms (lymphoid/hematopoietic) (%)	0.4	0.4	*p* = 0.08

**Table 5:** Comparison of hospitalization outcomes in propensity score-matched cohorts of unilateral TKA and bilateral TKA.

	Unilateral TKA	Bilateral TKA	Significance
Died during hospitalization	0.00%	0.02%	*p* < 0.001
Length of stay mean in days	2.49 (Std. deviation 1.3)	3.03 (Std. deviation 2.8)	*p* < 0.001
Total charges mean in USD	59,215 (Std. deviation 33,120)	83,639 (Std. deviation 52,551)	*p* < 0.001

## Data Availability

The original contributions presented in the study are included in the article/Supplementary Materials. Further inquiries can be directed to the corresponding author.

## Supplementary Materials

The following supporting information can be downloaded at https://www.mdpi.com/article/10.3390/healthcare13091033/s1: Table S1: Identification of Postoperative Complications Using Standardized ICD-10-CM Codes.

## Abbreviations

ASA	American Society of Anesthesiologists
B-TKA	Bilateral total knee arthroplasty
BMI	Body mass index
CCI	Charlson Comorbidity Index
CI	Confidence interval
CKD	Chronic kidney disease
DVT	Deep vein thrombosis
HCUP	Healthcare Cost and Utilization Project
ICD-10	International Classification of Diseases, 10th Revision
LOS	Length of stay
NIS	Nationwide Inpatient Sample
NSQIP	National Surgical Quality Improvement Program
OR	Odds ratio
PE	Pulmonary embolism
SPSS	Statistical Package for the Social Sciences
TKA	Total knee arthroplasty
U-TKA	Unilateral total knee arthroplasty
UTI	Urinary tract infection
